# Activating transcription factor 3 mediates apoptosis and cell cycle arrest in TP53-mutated anaplastic thyroid cancer cells

**DOI:** 10.1186/s13044-024-00202-x

**Published:** 2024-08-01

**Authors:** Abolfazl Kooti, Haniyeh Abuei, Alireza Jaafari, Shayan Taki, Jamileh Saberzadeh, Ali Farhadi

**Affiliations:** 1 Division of Medical Biotechnology, Department of Medical Laboratory Sciences, School of Paramedical Sciences, Shiraz University of Medical Sciences, Shiraz, Iran; 2 School of Medicine, Shiraz University of Medical Sciences, Shiraz, Iran; 3 Diagnostic Laboratory Sciences and Technology Research Center, School of Paramedical Sciences, Shiraz University of Medical Sciences, Shiraz, 7143918596 Iran

**Keywords:** ATF3, Mutant p53, TAp63, ΔNp63, SHARP1, Thyroid cancer, 8305C

## Abstract

**Background:**

It is believed that loss of p53 function plays a crucial role in the progression of well to poorly differentiated thyroid cancers including anaplastic thyroid carcinoma (ATC). Given the poor prognosis of ATC due to its strong therapeutic resistance, there is a need to establish new therapeutic targets to extend the survival of ATC patients. Activating transcription factor 3 (ATF3) can inhibit the oncogenic activity of mutant p53 and, as a result, contribute to tumor suppression in several TP53-mutated cancers. Herein, we demonstrate that the ectopic overexpression of ATF3 leads to the suppression of oncogenic mutant p53 activity in chemo-resistant 8305 C thyroid cancer cells harboring R273C p53 gene mutation.

**Methods:**

The biological behavior of 8305 C cells was assessed pre- and post-transfection with pCMV6-ATF3 plasmid using MTT assay, fluorescent microscopy, cell cycle, and annexin V/PI flow cytometric analysis. The effect of ectopic ATF3 overexpression on the cellular level of p53 was examined by western blotting assay. The mRNA expression levels of TP53, TAp63, ΔNp63, and SHARP1 were evaluated in ectopic ATF3-expressing cells compared to controls.

**Results:**

The overexpression of ATF3 in 8305 C thyroid cancer cells significantly decreased cell viability and induced apoptosis and cell cycle arrest in vitro. The immunoblotting of p53 protein revealed that ATF3 overexpression significantly increased the level of mutant p53 in 8305C cells compared to mock-transfected control cells. Additionally, elevated mRNA levels of TAp63 and SHARP1 and a decreased mRNA level of ΔNp63 were observed in PCMV6-AC-ATF3-transfected 8305 C cells with significant differences compared to the mock and untreated cells.

**Conclusion:**

In light of our findings, it is evident that therapeutic strategies aimed at increasing ATF3 expression or enhancing the interaction between ATF3 and mutant p53 can be a promising approach for the treatment of p53-mutated metastatic thyroid cancer.

**Supplementary Information:**

The online version contains supplementary material available at 10.1186/s13044-024-00202-x.

## Background

Anaplastic thyroid carcinoma (ATC) is a rare form of thyroid cancer (TC) comprising less than 2% of all TC cases. It appears to be one of the most aggressive human malignancies with a mean survival of 6 months postdiagnosis [1, 2]. Several therapeutic approaches are applied for ATC treatment including surgical resection combined with chemotherapy and radiotherapy. Moreover, anti-angiogenic agents, tyrosine kinase inhibitors, and hormone suppression therapy are a number of additional treatment options considered for ATC patients [3]. However, none of the established treatments available seem to prolong patient survival [4]. Alternatively, management of ATC has shifted from predominantly palliative and hospice care towards advanced molecular-based personalized therapies and surgery as warranted, leading to a nearly twofold increase in the overall survival rate and a threefold rise in survival compared to recently published data from single-institution studies [5]. Accordingly, development and introduction of novel molecular-based treatment protocols are of the essence.

p53 is a well-known tumor suppressor which is missing or mutated in almost half of all human malignancies [6]. Various mutations have been reported throughout p53 gene in different human cancers [7]. p53 gene mutations have been detected in over 80% of ATCs and play a major role in the progression of thyroid tumors from well differentiated, such as papillary and follicular, to poorly differentiated (anaplastic) TCs [8]. p53 mutations, particularly those occurring in “hot spots” such as R175H and R273H, cause not only p53 protein’s loss of tumor suppressor function, but also its acquisition of oncogenic activities including promoting cell proliferation, angiogenesis, and metastasis [9]. Previous studies have indicated that activating transcription factor 3 (ATF3) can bind to common mutant p53 protein and suppress its oncogenic activities [10]. The ability of mutant p53 to enhance cell migration and invasion could be linked to its interference with p63’s transcriptional activity, thereby preventing p63 from binding to its target genes including SHARP1 [11]. p63 is another transcription factor in p53 family of proteins whose expression is mediated by two alternative promoters, giving rise to two different isoforms, full-length TAp63 and N-terminally truncated ΔNp63 (Np63/DNp63). These two p63 isoforms perform not only different but sometimes opposing functions [12]. While TAp63 has been shown to induce apoptosis, ΔNp63 has been reported to inhibit the apoptotic function of TAp63, nominating TAp63 to be a tumor suppressor and ΔNp63 as a tumor promoter [13]. SHARP1 is a transcription factor with complex roles in apoptosis, cellular differentiation, and tumor progression [14]. This factor has been reported to be an important target of p63 protein whose suppression mimics mutant p53’s function of driving cell migration. Therefore, it seems that p63 exerts its tumor cell migration- and metastasis-inhibiting activities through SHARP1 [15]. There is evidence that ATF3 can suppress the oncogenic activity of mutant p53 by directly binding to it and altering its conformation so that it can no longer interact with p63 to inactivate it [10, 16]. Accordingly, p63 remains active and continues its tumor suppressor activities including the expression of SHARP1 [10].

ATF3, a member of the ATF/cAMP response element-binding (CREB) family of transcription factors, has recently been proposed by several studies as a new tumor suppressor factor. The anti-tumor activity of ATF3 has been established in glioblastoma, bladder, lung, colon, and cervical cancer [17–23]. However, other studies have reported that ATF3 can promote tumor growth and metastasis in breast cancer and cutaneous squamous cell carcinoma [24, 25]. Therefore, it seems that ATF3 can play opposing roles in the context of tumorigenesis While Xiao et al. have recently investigated the impact of ATF3 overexpression on the behavior of thyroid cancer cells and demonstrated ATF3 as a tumor suppressor gene in thyroid cancer progression [26], the effect of ectopic ATF3 expression on the biological behavior of the chemo-resistant 8305 C thyroid cancer cell line, representative of undifferentiated ATC harboring the R273C p53 gene mutation, has not yet been examined regarding the expression of metastasis-related genes, proliferation, apoptosis, and cell cycle analysis.

## Methods

### ATF3 gene cloning in pCMV6 vector

The ATF3 gene was synthesized and cloned in the pUC57 vector by BIOMATIK Company according to the ATF3 mRNA sequence from NCBI (NM_001674.3). The pUC57 vector was then utilized as a template for ATF3 amplification by polymerase chain reaction (PCR) using pfu polymerase (Sigma-Aldrich) and subsequently cloned in the pCMV6-AC-IRES-GFP vector as described elsewhere [23]. *E. coli* DH5α (Pasteur Institute, Tehran, Iran) competent cells were transformed with the pCMV6-ATF3 recombinant plasmids. Subsequently, the construction of the cloned vector was confirmed by colony PCR, enzyme digestion, and Sanger sequencing. Following amplification in DH5α cells, the recombinant plasmids were purified using FavorPrep Plasmid DNA Extraction Mini Kit (Favorgen, Taiwan) according to the manufacturer’s instructions.

### Cell culture and transfection

8305 C cells (NCBI Code: C597, purchased from Pasteur Institute, Tehran, Iran) were cultured in a 5% CO2 atmosphere at 37 °C, in complete DMEM (high glucose) with 10% fetal bovine serum (FBS), 2mM L-glutamine, and 1% penicillin/streptomycin. After 24 h, cells were transfected with the recombinant plasmids by calcium phosphate method. To this end, 8 × 10^5^ 8305 C cells were seeded in 6-well cell culture plates. Once cells reached 70–75% confluence, the medium was replaced with fresh DMEM. After 2 h, the cells were incubated with calcium phosphate-precipitated DNA (2–7 µg of DNA, 25 µL of 2.5 M CaCl2 solution, and 200 µL of 2× HEPES-buffered saline solution) for 15 h. Additionally, a second group of 8305 C cells was transfected with the empty vector (mock plasmid), and a third group received only calcium phosphate transfection reagents, without any plasmid. Cells were then washed with PBS three times and cultured in a fresh complete growth medium for 48 and 72 h.

### Fluorescence microscopy and flow cytometry analysis

Transmitted light microscopy and fluorescence microscopy (Olympus, Tokyo, Japan) were applied to evaluate transfection efficiency 24, 48, and 72 h post-transfection. At each time point, four images were captured and examined individually. Transfection efficiency was calculated by counting green cells in a total of 100 cells per image. Furthermore, the cells were washed with PBS and then detached from the cell culture plate using trypsin-EDTA solution 48 and 72 h post-transfection for flow cytometry analysis. Following detachment, the cells were centrifugated at 350 g for 5 min and then resuspended in PBS and examined for fluorescent intensity with a FACSCalibur flow cytometry analyzer (BD Biosciences; San Jose, California, USA). In each experiment, a total of 10^4^ cells were considered for analysis. Finally, the data were assessed using FlowJo software version 10.0 (FlowJo LLC, Ashland, OR, USA).

### MTT assay

In order to evaluate the cytotoxicity of pCMV6-ATF3 plasmid, MTT assay was employed. Briefly, 8305 C cells were seeded in 96-well plates at the density of 9,000 cells per well in 200 µL of DMEM containing 10% Fetal Bovine Serum (FBS) and 1% Pen/Strep, and incubated at 37 °C with 5% CO2. Once the cells reached 70% confluence, calcium phosphate-precipitated DNA (0.1–2 µg of pCMV6-ATF3) was added to the wells according to the above-mentioned transfection protocol. Cell viability was examined 24, 48 and 72 h post-transfection by adding 200 µL of fresh medium containing 20 µL of MTT solution (5 mg/mL) (Sigma-Aldrich) to each well, followed by a 4-hour incubation. Finally, 150 µL of DMSO (Sigma-Aldrich) was added to each well and the absorbance was measured at 570 nm with a Sat Fax 2100 microplate reader (Awareness Technology Inc., Palm City, FL, USA). The MTT assay was conducted in triplicate.

### Cell cycle analysis

8305 C cells were seeded in 6-well plates at a density of 2 × 10^5^ cells per well and transfected with 6 µg of pCMV6-ATF3 or mock plasmids. After 48 and 72 h, the cells were harvested and fixed with cold 70% ethanol at 4 °C for 1 h. Afterwards, the cells were washed with PBS twice and stained with 20 mg/mL PI solution (Sigma-Aldrich) containing 0.1 mg/mL RNase A (Sigma-Aldrich) for 30 min at room temperature in the dark. Cell cycle analysis was carried out by flow cytometry. A total of 20,000 cells were examined per group. The data were analyzed using FlowJo software version 10.0.

### Apoptosis assay

The apoptosis level was examined in 2 × 10^5^ transfected 8305 C cells. Briefly, cells were washed with PBS, detached from the plate, and collected by centrifugation 48 and 72 h post-transfection. The cells were washed twice with PBS and subjected to apoptosis assay using a PE-Annexin V Apoptosis Detection Kit (BD Biosciences, Bedford, MA, USA) according to the manufacturer’s protocol. In brief, 6 µg of pCMV6-ATF3 or mock transfected 8305 C cells along with untreated cells were resuspended with 400 µL binding buffer and incubated with 5 µL PE-Annexin V for 15 min in the dark. Next, 5 µL 7-AAD was added to the cell suspension, followed by another 15-min incubation at room temperature in the dark. Finally, flow cytometric analysis was performed with a Flow Cytometer FACS Calibur and the data were further analyzed using FlowJo software version 10.0.

### Cell lysis and protein assay

8305 C cells were seeded in 6-well culture plates and incubated overnight at 37 °C with 5% CO2. Subsequently, the cells were transfected following the aforementioned procedure. At 48 and 72 h post-transfection, cells were detached and washed twice with cold PBS. The cell suspension was then sonicated (Amplitude: 80–100, 2 cycles of 30 s each with 30-second cooling intervals) in lysis buffer (50 mM Tris-HCl, 10 mM EDTA, 1% SDS, pH 8.0) supplemented with a protease inhibitor cocktail. Samples were centrifugated at 15,000 g for 20 min at 4 ℃, and the supernatants were collected. The protein concentrations of both control and test samples were measured using the Bradford protein assay and a standard curve of absorbance was generated.

### ATF3 and p53 western blotting analysis

The cell lysate was mixed with 5× sample buffer and subsequently boiled at 95 °C for 10 min. Afterwards, the samples were subjected to SDS-PAGE based on the method of Laemmli [27] for western blot analysis. Briefly, equal amounts of proteins from each sample underwent electrophoresis in 12% SDS-polyacrylamide gels and then transferred onto polyvinylidene fluoride (PVDF) membranes (Millipore, Feltham, United Kingdom) using Bio-Rad Mini Trans-Blot® electrophoretic transfer cell at 300 mA for 3 h. Next, membranes were incubated in a blocking solution (5% skim milk, 0.05% Tween 20) for 16 h and then washed with TBST buffer (150 mM NaCl, 50 mM Tris-base, and 0.05% Tween 20) for 15 min. Afterwards, the abundance of ATF3 and p53 protein was measured using monoclonal anti-ATF3 and anti-p53 antibodies (1:500, Santa Cruz Biotechnologies, Santa Cruz, CA, USA), along with anti-β-actin antibody (dilution, 1:5000, Santa Cruz Biotechnology) as an internal control. After three rounds of washing with TBST, horseradish peroxidase-conjugated secondary antibody (1:10000, Sigma-Aldrich) was added to the membrane, followed by a 1-hour incubation at room temperature and then two rounds of washing with TBS buffer (50 mM Tris-base and 150 mM NaCl). Eventually, 10 mL of substrate solution containing 5 mg of 3,3′-Diaminobenzidine (DAB) (Sigma-Aldrich) and 5 µL H_2_O_2_ in 10 mL of DDW was added to the PVDF surface. DDW was added to stop the reaction.

### Reverse transcription quantitative PCR (RT‑qPCR)

The oligonucleotide primer sequences used for RT-qPCR were designed using Gene Runner 3.0 software (http://www.generunner.net/) and are presented in Table 1 along with their corresponding annealing temperatures. Total RNA was extracted from cells using the RiboEx TM kit (GeneAll, Korea) and reverse transcribed to cDNA at 37˚C for 15 min using the PrimeScript RT reagent kit (TaKaRa, Japan). RT-qPCR was performed with an ABI Prism 7000 Sequence Detection System (Applied Biosystems) using RealQ Plus 2x Master Mix Green (Ampliqon, Denmark). To determine primer efficiency, a standard curve was generated for each set of primers. The relative fold changes of the desired genes were calculated according to the Pfaffl method [28] and GAPDH was used as a housekeeping gene (Forward: GGCCTCCAAGGAGTAAGACC, Reverse: AGGGGTCTACATGGCAACTG) [29]. The thermocycling conditions were as follows: Initial denaturation at 95˚C for 15 min, followed by 40 cycles of 95˚C for 15 s, annealing temperature for 30 s, and 72˚C for 30 s.

**Table 1:** Oligonucleotide primer sequences used for reverse-transcription real-time PCR assay

Primer name	Sequences (5’ to 3’)	PCR product size (base pair)	Annealing temperature (°C)
ATF3-Forward	GAGTGCCTGCAGAAAGAGT	117	58
ATF3-Reverse	CCGATGAAGGTTGAGCATG		
p53-Forward	ATGAGCGCTGCTCAGATAG	156	57
p53-Reverse	TGGTACAGTCAGAGCCAAC		
TAp63-Forward	CTCATGCAGTACCTTCCTC	117	56.5
TAp63-Reverse	CACAAGCTCATTCCTGAAG		
ΔNp63-Forward	CAATGCCCAGACTCAATTTAG	108	56
ΔNp63-Reverse	GTGTTATAGGGACTGGTGG		
SHARP1-Forward	GAGACGACACCAAGGATAC	140	56
SHARP1-Reverse	CTCCAGATGTCCCAGAGTTG		

### Statistical analysis

Data analysis was performed using Statistical Package for the Social Sciences (SPSS) software version 22, and the results were presented as mean ± SD. As the data did not follow a normal distribution, the Kruskal-Wallis statistical test was employed to assess the significant differences between the groups. *P* < 0.05 was considered statistically significant.

## Results

### Transfection of pCMV6-ATF3 vector into 8305 C cells

**Fig. 1:**
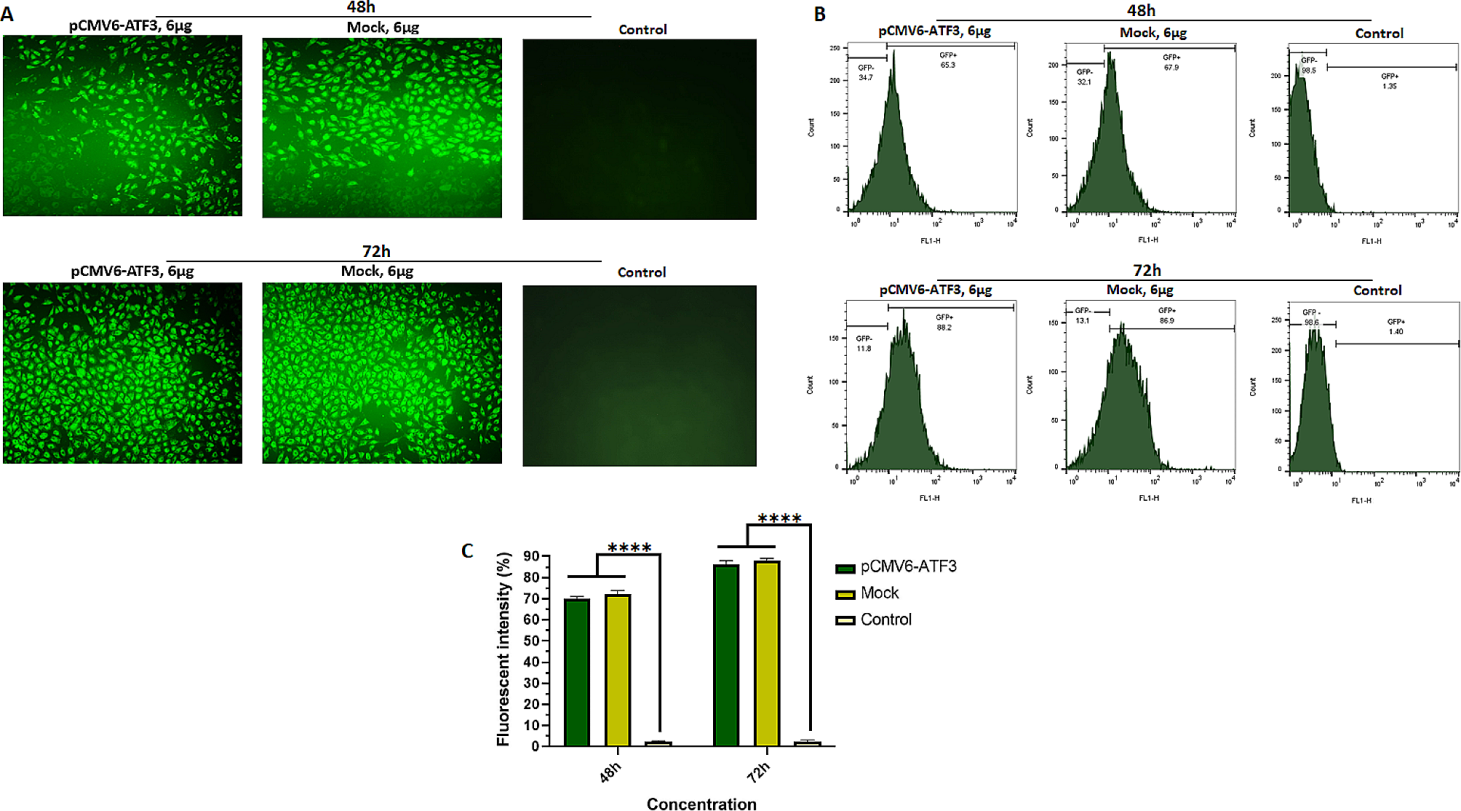
Fluorescent intensity for pCMV6-ATF3-tranfected, mock-transfected, and control 8305 C cell groups using fluorescence microscopy and flow cytometry. (A) 8305 C cells transfected with 6 µg of the pCMV6-ATF3, empty vector (mock), and control group (8305 C cells were cultured under the same conditions, but without transfection agents). Cells were observed using a 20-fold objective lens. (B) Histograms from FACS analysis for 6 µg of pCMV6-ATF3, mock-transfected, and control group. (C) Average fluorescent intensity for pCMV6-ATF3, mock-transfected, and control group. Flow cytometry analysis showed that the average transfection rates for pCMV6-ATF-transfected cells after 48 and 72 h were 69.7% and 86.6%, respectively. **** *P* ≤ 0.0001

To enhance the transfection efficiency of the recombinant plasmids into 8305 C cells, different amounts of plasmid DNA were used for the calcium phosphate transfection method. Increasing the amount of DNA for transfection led to a higher rate of GFP fluorescence-positive cells. However, the transfection efficiency plateaued when the amount of DNA exceeded 6 µg. Additionally, the fluorescence emitted by the transfected cells reached its peak 72 h post-transfection. Consequently, based on the ratio of the number of cells to the amount of DNA (see Additional File Table S1), the experimental conditions were established as follows: 6 µg of plasmid DNA was used for transfection, and the transfected cells were analyzed 72 h post-transfection. Images of the transfected cells were captured using fluorescent and transmitted light microscopy (Fig. 1A). The images were manually analyzed, and the transfection efficiency was quantified by calculating the ratio of GFP fluorescence-positive cells to the total cells.

Our results indicated a transfection efficiency of over 80% for both pCMV6-ATF3 and mock plasmids. Furthermore, the average transfection rates of 69.7% and 86.6% were observed for pCMV6-ATF3 vectors by flow cytometry at 48 and 72 h post-transfection, respectively, as presented in Fig. 1B and C.

### ATF3 suppresses the proliferation/viability of 8305 C cells

**Fig. 2:**
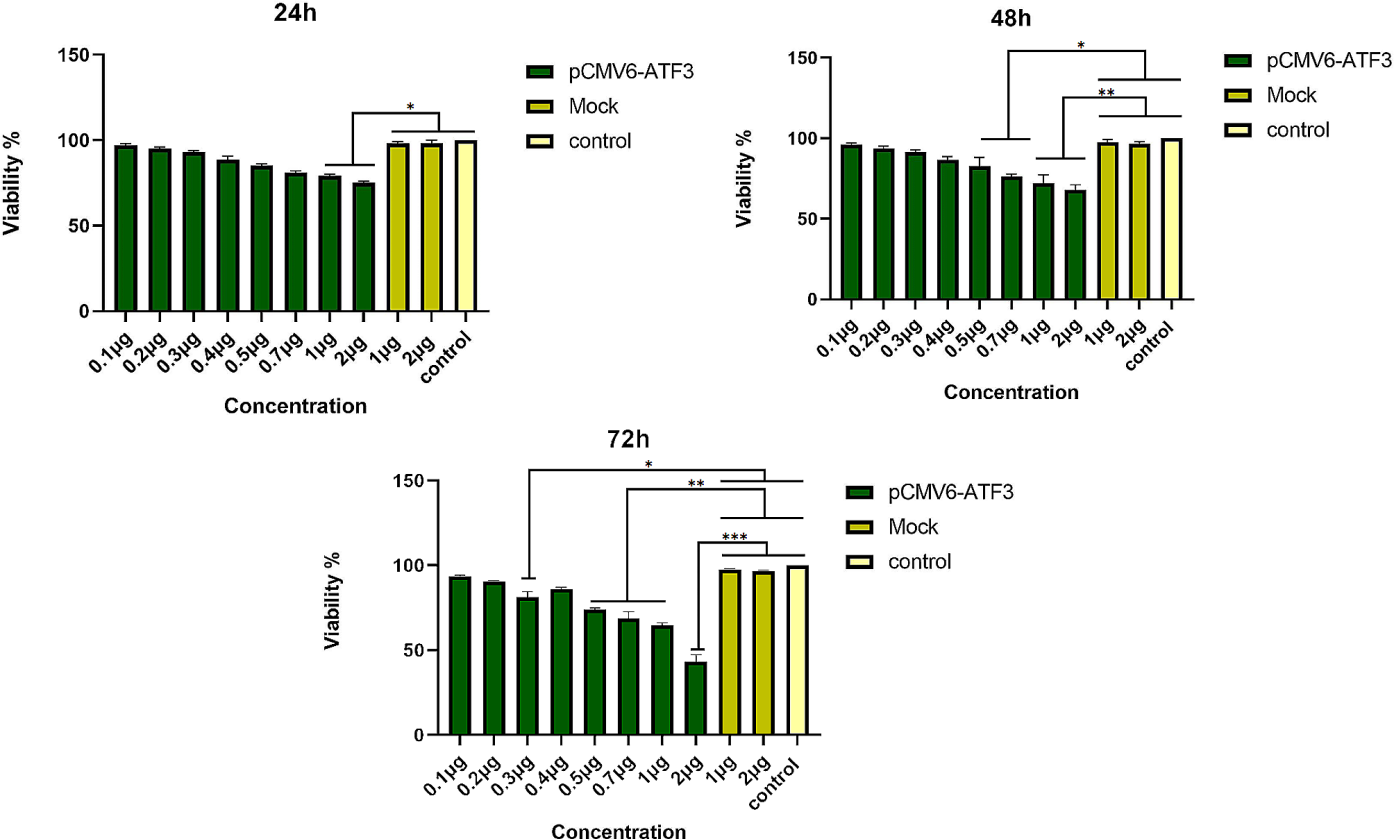
8305 C cells were transfected with varying concentrations of the pCMV6-ATF3 plasmid (ranging from 0.1 to 2 µg), and their viability was evaluated by MTT assay after 24, 48, and 72 h. * *P* ≤ 0.05, ** *P* ≤ 0.01, *** *P* ≤ 0.001

The MTT assay was used to assess the viability of 8305 C cells following transfection with pCMV6-ATF3. Cells were treated with pCMV6-ATF3 at a series of DNA concentrations (0.1–2 µg) and mock (1 and 2 µg) for 24, 48 and 72 h. MTT assay revealed that ATF3 overexpression at a concentration of 2 µg resulted in the maximum inhibition of 8305 C cell growth (61% at 72 h), which was significantly higher than that of untreated cells and mock-transfected ones (*p* < 0.001) as illustrated in Fig. 2.

### ATF3 mediates cell cycle arrest and apoptosis in 8305 C cells

**Fig. 3:**
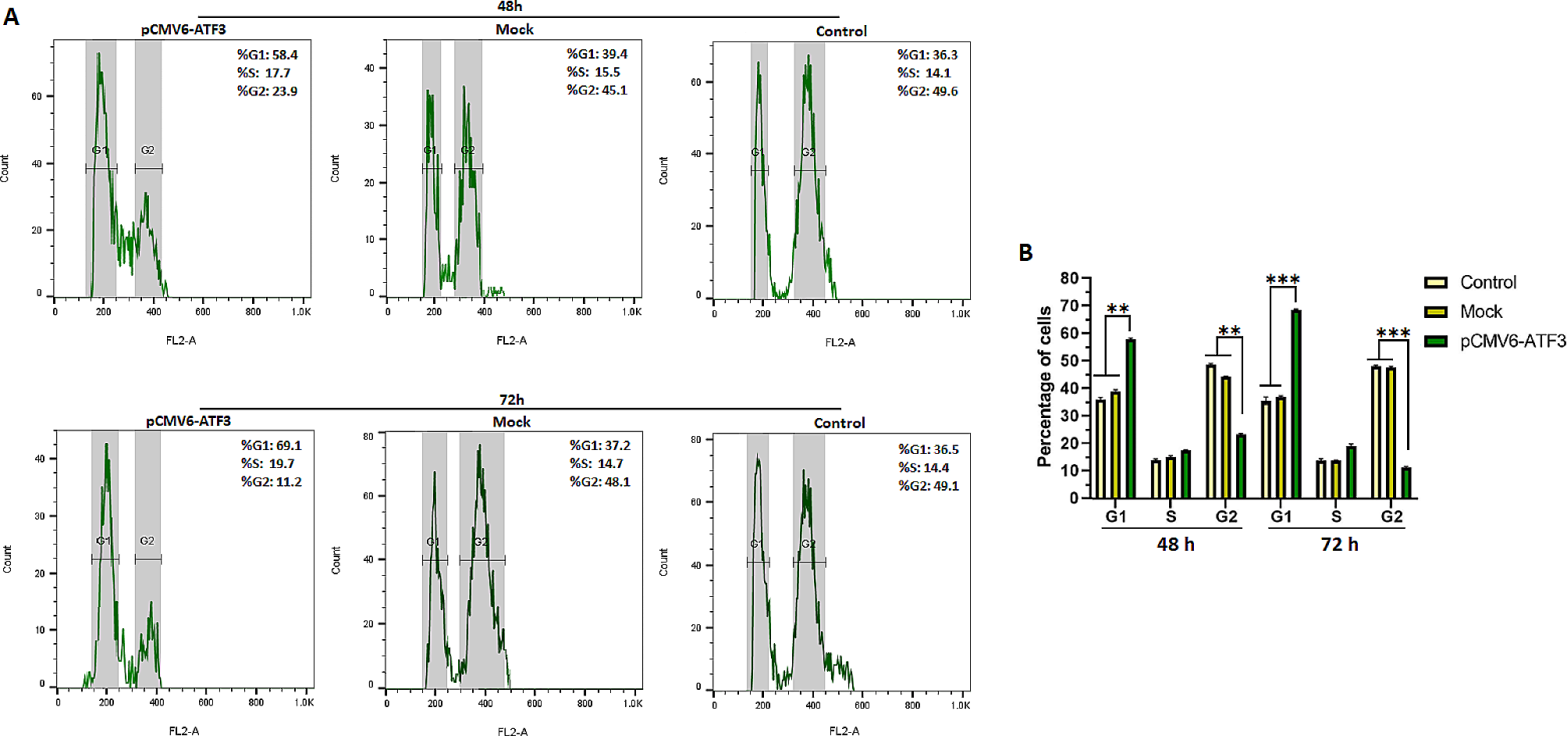
Cell cycle analysis results. The cell cycle histogram represents the percentage of cells in the G1, S, and G2/M phases at 48 and 72 h post-transfection (A), compared to mock and control groups (*p* < 0.0006). The distribution of cells in the G1, S, and G2/M phases at 48 and 72 h post-transfection (B). No significant difference was observed between the mock and control groups (*p* > 0.05). ** *P* ≤ 0.01, *** *P* ≤ 0.001

Following ATF3 overexpression, a rise was observed in the percentage of cells in the G1 phase and, to a lesser degree, those in the S phase. On the other hand, the proportion of cells in the G2/M phase decreased (Fig. 3). Our findings indicate that cell cycle was significantly blocked in the G0/G1 phase in 8305 C cells transfected with pCMV6-ATF3 plasmids compared to the mock and control groups (*p* < 0.0006). Furthermore, no significant alteration in the cell cycle was found in the mock plasmid-transfected 8305 C cells compared to the control groups (*p* > 0.05).

**Fig. 4:**
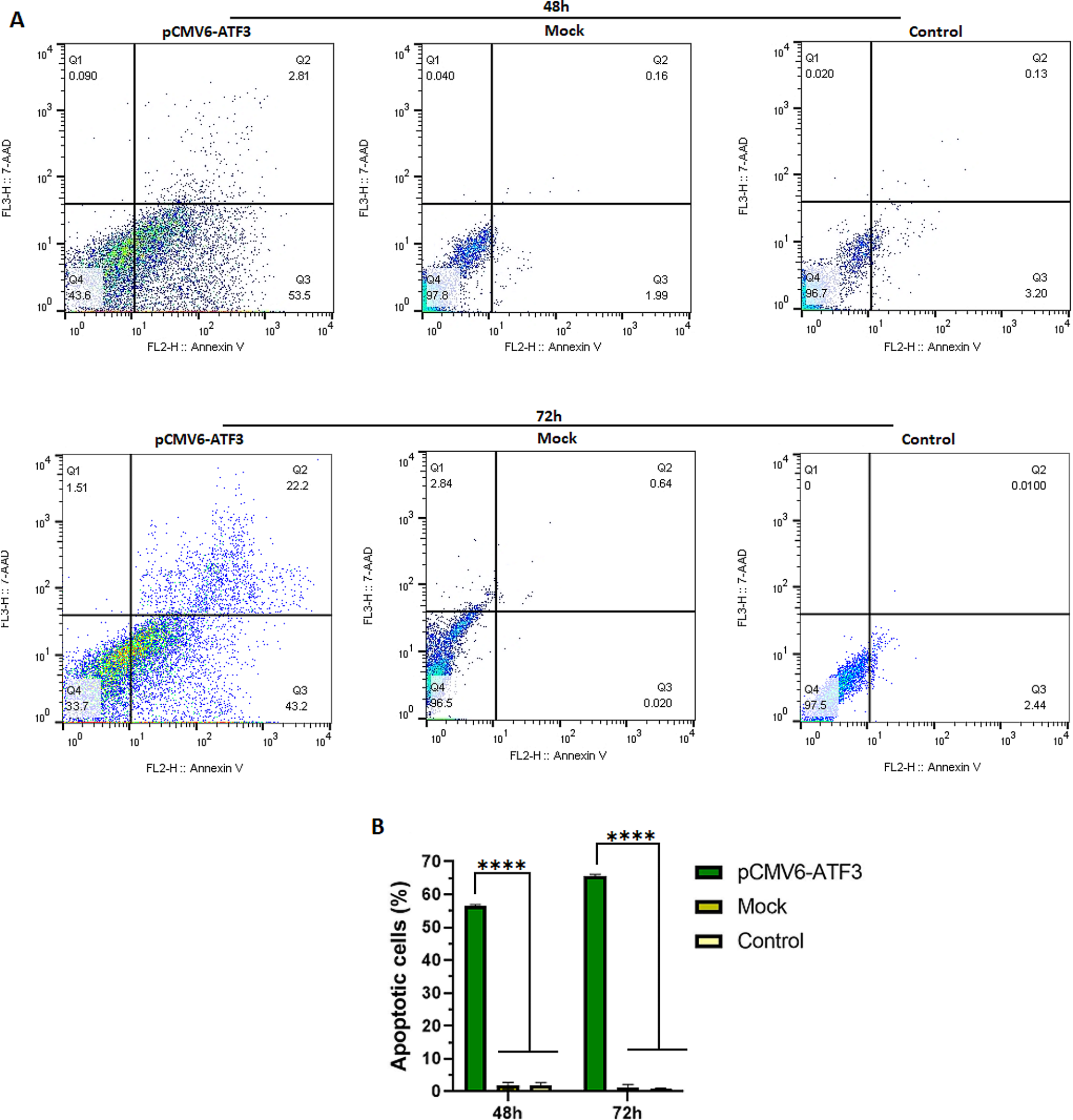
Apoptosis induced by ATF3 overexpression in 8305 C cells. The histogram represents the percentage of total apoptotic cells in different groups. FlowJo software version 10.0 was utilized to generate the flow cytometry plots. 8305 C cells were transfected with 6 µg of pCMV6-ATF3 prior to the measurement of apoptosis level using the PE Annexin V apoptosis detection kit after 48 and 72 h (A). A statistically significant increase in the number of apoptotic 8305 C cells was observed compared to the untreated and mock groups (*P* < 0.0001). However, the difference in the number of apoptotic cells between the untreated and mock groups was not significant (*p* > 0.05) (B). Q1, Q2, Q3, and Q4 indicate necrotic, late apoptotic, early apoptotic, and viable cells, respectively. The percentage of total apoptotic cells was calculated from Q2 + Q3. **** *P* ≤ 0.0001

To investigate the apoptotic effect of ATF3, 8305 C cells transfected with pCMV6-ATF3 and mock plasmids were examined by flow cytometry at 48 and 72 h post-transfection. As presented in Fig. 4, our results demonstrate that transfection with pCMV6-ATF3 significantly increased the apoptosis rate in 8305 C cells (*p* < 0.0001), up to 55.7 ± 1% and 65.8 ± 1% at 48 and 72 h post-transfection, respectively (Fig. 4).

### Effect of ATF3 overexpression on the expression of p53, TAp63, ΔNp63, and SHARP1

**Fig. 5:**
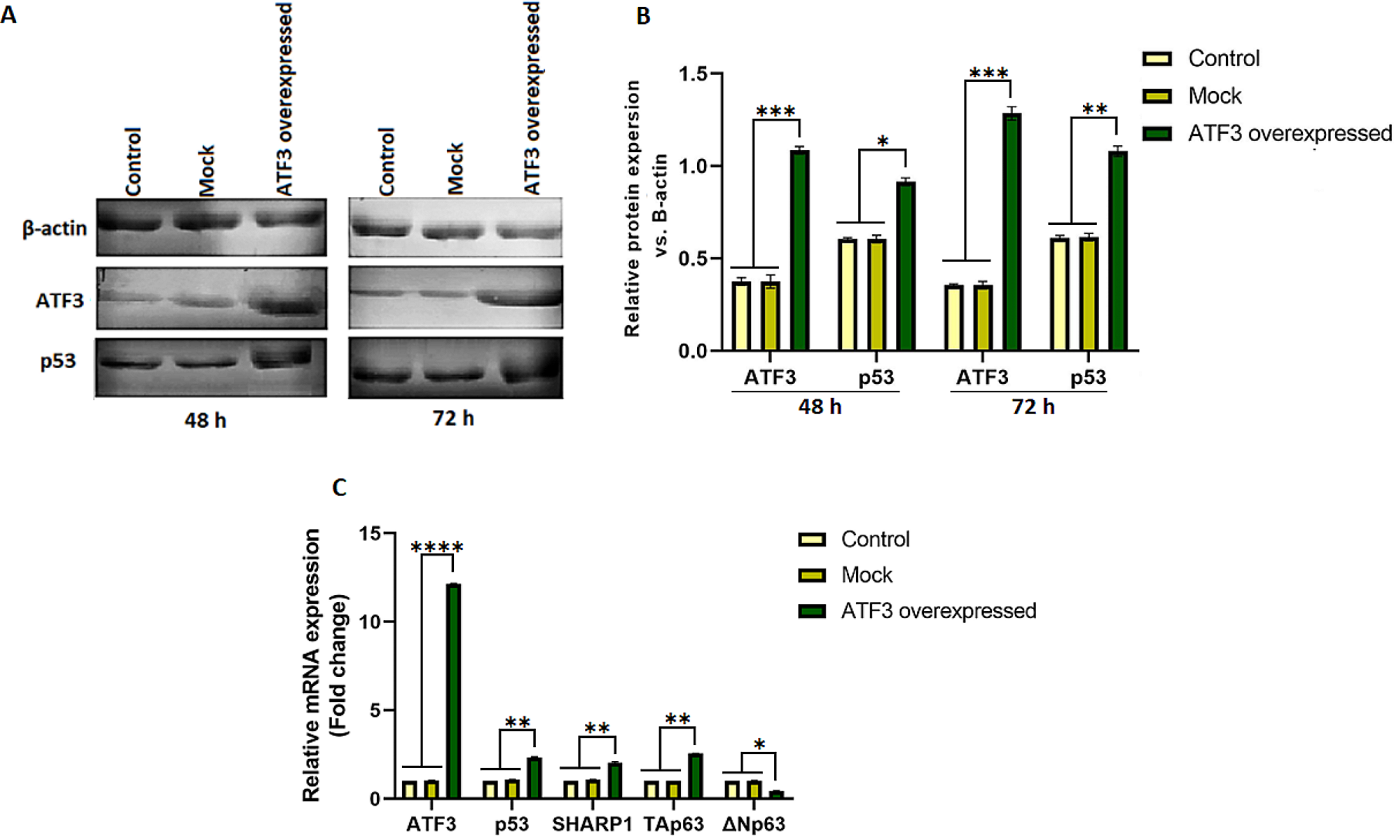
ATF3 and p53 protein levels in 8305 C cells were determined by western blotting. Whole cell lysates were subjected to western blotting with anti-ATF3, anti-p53, and anti-β-actin antibodies. (A) 8305 C cells were transfected with pCMV6-ATF3 and mock plasmids at a concentration of 6 µg, and untreated cells were included after 48 and 72 h. (B) Densitometry analysis of relative protein expression levels after 48 and 72 h. Higher expression of ATF3 was observed in 8305 C cells transfected with pCMV6-ATF3 plasmids compared to the untreated and mock groups (*p* < 0.0008). The results of western blotting also demonstrated that the overexpression of ATF3 had a significant effect on p53 levels in 8305 C cells compared to the untreated and mock-transfected cells (*p* < 0.002). (C) ATF3 overexpression led to an increase in p53, TAp63, and SHARP1 gene expression (*p* < 0.001), and a decrease in ΔNp63 gene expression (*p* < 0.01) in comparison with the mock and control groups. * *P* ≤ 0.05, ** *P* ≤ 0.01, *** *P* ≤ 0.001, **** *P* ≤ 0.0001

The overexpression of ATF3 in transfected 8305 C cells was confirmed by western blot analysis. In addition, the expression levels of ATF3, p53, and β-actin proteins were evaluated in untreated, mock-transfected, and pCMV6-ATF3-transfected groups. As displayed in Fig. 5, only low levels of ATF3 were detectable in the untreated and mock-transfected cell groups, whereas a significantly higher level of ATF3 expression was observed in pCMV6-ATF3-transfected cells (*p* < 0.0008). The results of western blotting also revealed a significantly increased level of p53 protein in 8305 C cells transfected with pCMV6-ATF3 plasmids compared to the control cell groups (*p* < 0.002).

At the mRNA level, RT-qPCR analysis 72 h post-transfection revealed that increased ATF3 gene expression in 8305 C cells transfected with pCMV6-ATF3 plasmid caused significantly elevated p53, TAp63, and SHARP1 gene expression (*p* < 0.002). On the other hand, ATF3 overexpression led to a decrease in ΔNp63 gene expression (*p* < 0.02) in comparison with the control and mock groups. The results of the changes of gene expression in the transfected groups by pCMV6-ATF3, mock, and control are summarized in Fig. 5C.

## Discussion

ATC is rare but comprises more than 90% of all endocrine cancers. It is extremely aggressive, has a poor prognosis due to its high therapeutic resistance, and accounts for up to 30–40% of thyroid cancer-related mortality [30]. While there are ongoing efforts to develop new treatments for ATC, there remains a lack of efficient therapeutic options for advanced local or metastatic ATC. Hence, it is necessary to elucidate the molecular mechanisms behind the progression and metastasis of TC in order to develop novel treatment approaches. A newly released study investigating the role and regulation of ATF3 in thyroid cancer revealed that DNA hypermethylation occurring at the CpG islands of the ATF3 promoter results in the blockage of SP1 and MYC-MAX binding to the promoter region and consequently the suppressed expression of the ATF3 gene. Further, this reduction in ATF3 expression likely contributes to the progression of thyroid cancer by directly regulating prognostic genes within the MAPK and PI3K/AKT pathways [26]. The current study contributes to our comprehension of the anticancer activities of ATF3, an aberrantly expressed protein in a variety of human malignancies [31]. Herein, we have demonstrated for the first time that ATF3 acts as a tumor suppressor factor that inhibits cell proliferation and mediates apoptosis and cell cycle arrest in vitro. Initially, we demonstrated the successful overexpression of ATF3 in 8305 C cells following their transfection with pCMV6-ATF3 vector. The results of the cell cycle analysis indicated that ATF3 overexpression led to a significant blockage of the cell cycle in the G0/G1 phase. This suggests that ATF3 might be involved in inhibiting the progression of cells from G1 to S phase. Lack of significant cell cycle alteration in mock plasmid-transfected cells suggests that the observed effects are specific to the overexpression of ATF3. Moreover, it is well-known that p53 interrupts the progression of the cell cycle in the G0/G1 phase by elevating the expression of p21/CDKN1A which inhibits the activity of cyclin D and CDK4/6 [32]. Accordingly, we examined whether ATF3 induces cell cycle arrest through the p53 pathway. Our results indicated that the overexpression of ATF3 in 8305 C cells increased apoptosis significantly compared to the mock-transfected and untreated groups. Furthermore, the immunoblotting of p53 protein revealed that ATF3 overexpression caused a significant increase in the level of mutant p53 in the 8305 C cells. Previous studies also suggest an association between ATF3 and p53 regarding the involvement of ATF3 in the regulation of tumor suppressor activities; however, they argue that the elevation of p53 level does not occur through ATF3’s transcriptional activity, but rather through the direct interaction of ATF3 with p53 protein, resulting in the stabilization of p53 and maintenance of its tumor suppressor activity [31, 33]. Furthermore, findings of another study show that ATF3 can bind to and counteract common mutant p53 protein with the same affinity as that of its binding to the normal p53 protein and accordingly, it can reverse the drug resistance caused by p53 mutation and inhibit the migration of R175H-expressing SKBR3 and R273H-harbouring A431 cancer cell lines [10]. Given that ATF3 binds to the C terminus of p53 which is often not affected by p53 mutations [33], it is probable that ATF3 could interact with many of mutant p53 proteins and modulate their oncogenic activities. On the other hand, a number of studies have reported that ATF3 promotes the trans-activation of p53 gene, thereby increasing its expression in response to various oncogenic stresses [34–36]. The results of our study also confirm that ATF3 plays a part in p53 gene expression. Interestingly, a previous study has demonstrated that p53 can directly activate the expression of ATF3 gene as one of its target genes [37], indicating a functional link between p53 and ATF3. In addition, the role of other factors including FOXP3, a key downstream mediator of p53-induced cellular senescence [38] which has recently been shown to down-regulate the expression of ATF3 protein by reducing the activity of ATF3 promoter [36] should be taken into consideration to dissect the p53-ATF3 interactions and discover their potential regulatory loops and mechanisms.

A key mechanism of the mutant p53’s oncogenic activity occurs through its ability to bind to another member of the p53 family, p63, and disrupt its tumor suppressor functions. In order to further elaborate on the effect of ATF3 on the mutant p53 functions, we next examined the effect of ATF3 overexpression on full-length TAp63, N-terminally truncated ΔNp63, and SHARP1 gene expression. While TAp63 (transcription-competent TA) transactivates multiple p53 target genes resulting in cell cycle arrest and apoptosis [39], ΔNp63 (transcription-deficient ΔN) which does not possess the N-terminal transactivation domain present in TAp63, acts in a dominant-negative manner to disrupt the activity of p53, TAp63 and TAp73 [40]. Generally, ΔNp63 is overexpressed in many human cancers while TAp63 is considered to be a tumor suppressor protein [41]. In contrast, Malaguarnera et al. have previously reported that p63 may contribute to the progression of TC [42]. They carried out co-immunoprecipitation assays on two p63-positive TC cell lines, including C-643 that harbors K248Q and K286E p53 mutations and FTC-133 with R273H p53 mutation, as well as a p63-negative TC cell line, C-98, expressing a mutant p53 version with K286E mutation and concluded that p53 mutants expressed in TC cell lines do not interact with p63. However, co-immunoprecipitation experiments were not conducted on 8305 C cells which carry a distinct p53 mutation, R273C.

It is believed that the interaction between mutant p53 and TAp63 can inactivate the transcriptional activity of TAp63 by preventing TAp63 from binding to its target genes including SHARP1, the principal suppressor of cancer cell migration and metastasis [43, 44]. In our study, elevated TAp63 mRNA and decreased ΔNp63 mRNA levels were observed in pCMV6-ATF3 transfected 8305 C cells with a significant difference compared to the mock and untreated groups. Our work uniquely demonstrates a role for ATF3 causing high gene expression of TAp63. Given that p53 and p63 share a similar arrangement of exons and introns, along with a significant similarity in their amino acid sequence, particularly in their central DNA-binding domains [45], and considering the fact that ATF3 binds to multiple regions of the genome where p53 is already attached [46], it can be hypothesized that ATF3 can bind to and positively regulate the transcriptional activity of TAp63 gene. However, additional research to investigate the interplay between ATF3 and TAp63 within the genome is required.

**Fig. 6:**
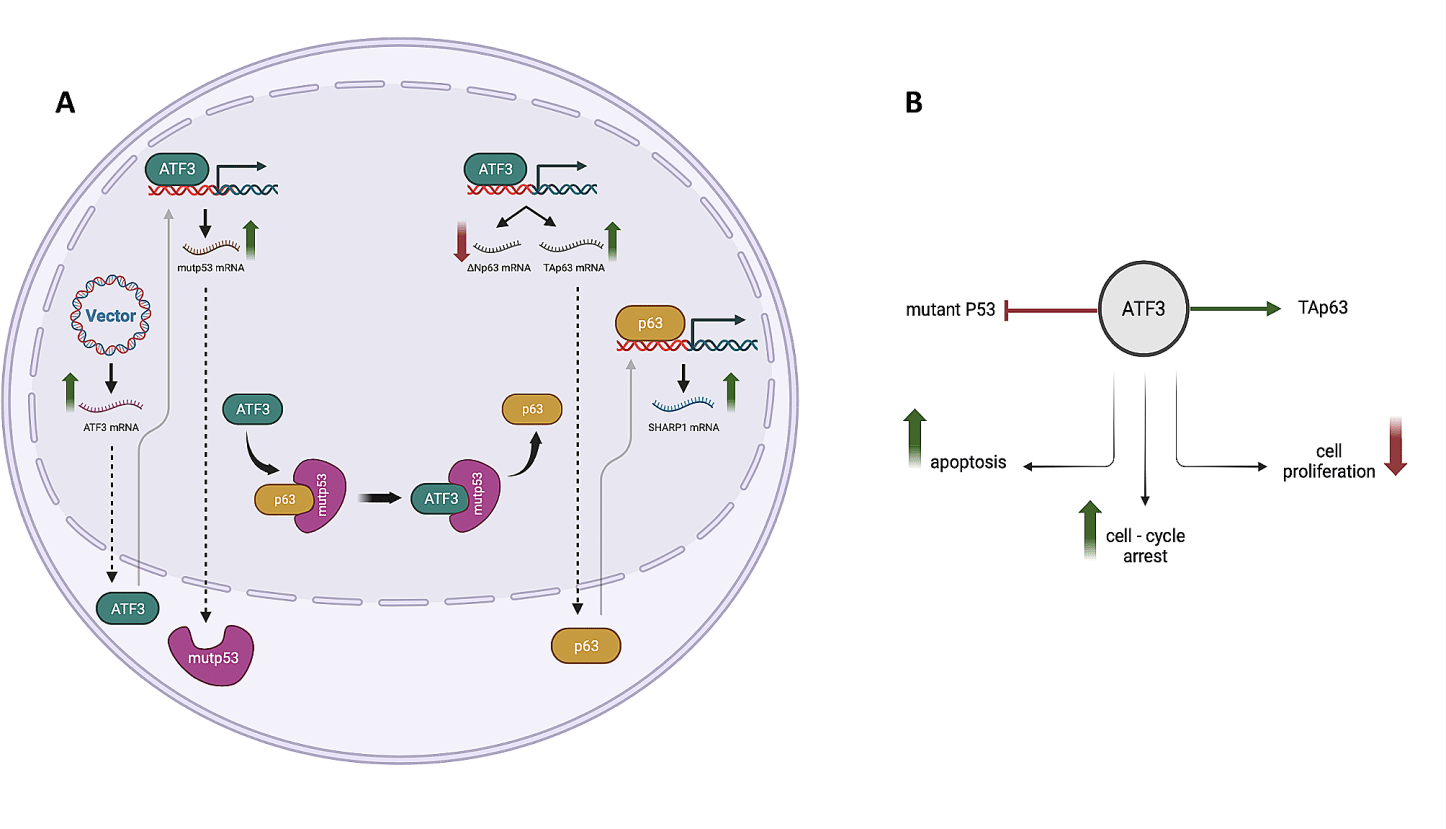
(A) Upon transfection with PCMV6-ATF3 plasmids, the overexpressed ATF3 mediates the transcription of p63 and mutant p53 genes and interferes with the interaction between p63 and mutant p53 proteins, resulting in the reactivation of p63 transcriptional activity and the expression of its target genes, such as SHARP1. (B) ATF3 regulates apoptosis, cell cycle arrest, and cell proliferation in TP53-mutated 8305 C ATC cells. Figure was created with BioRender.com

Of note, our data provided evidence that ATF3 significantly increased the mRNA expression of a p63-target gene, SHARP1 in mutant p53-expressing cells. Wei et al. have reported that the mutant ATF3 was not able to prevent mutant p53 from binding to and interacting with TAp63 leading to the reactivation of p63 possibly by direct binding to p63, thereby inhibiting the oncogenic function of mutant p53 and sensitizing p53 mutant cancer cells to anti-cancer medications [10]. With this in mind, it seems logical for ATF3 to reactivate the transcriptional activity of p63 and, accordingly, increase SHARP1 mRNA in mutant p53-expressing cells. SHARP1 is known for its ability to inhibit cancer cell invasion, migration, and metastasis (Fig. 6). Findings from a previous study have shown that the overexpression of SHARP1 leads to a significant suppression of the invasion and migration of TPC-1 and TT thyroid cancer cells [47]. Due to its anti-proliferative role in human TC which is consistent with the findings of the present study, SHARP1 can be a potentially valuable target for therapeutic interventions. The limitation of the present study is that all experiments were conducted solely in 8305 C cells, which may not suffice for drawing comprehensive conclusions. Further studies involving various thyroid cancer-derived cell lines are imperative to validate the functional interplay among ATF3, mutant p53, TAp63, ΔNp63, and SHARP1 and their significance in conferring drug resistance in TCs. In addition, coimmunoprecipitation studies are recommended to investigate the interaction between ATF3, p53, and p63.

## Conclusions

To sum up, our results suggest that ATF3 can bind to R273C mutant p53 in chemo-resistant 8305 C thyroid cancer cells and disrupt the interaction between mutant p53 and p63. Accordingly, while mutant p53 interferes with the transcriptional activity of p63, ATF3 has the ability to counteract mutant p53 and restore the normal functioning of p63. In conclusion, our findings suggest that ATF3 can suppress mutant p53 gain-of-function and could hold promise in developing novel therapeutic strategies for p53-mutated ATC that are aimed at increasing ATF3 expression or enhancing the interaction between ATF3 and mutant p53.

### Electronic supplementary material

Below is the link to the electronic supplementary material.

Supplementary Material 1

## Data Availability

All data generated or analysed during this study are included in this published article and its supplementary information file.

## Abbreviations

ATC: Anaplastic thyroid carcinoma
ATF3: Activating transcription factor 3
TC: Thyroid cancer
