# Supplementary material for: Activating transcription factor 3 mediates apoptosis and cell cycle arrest in TP53-mutated anaplastic thyroid cancer cells
Source: Thyroid Res. 2024 Aug 1;17:12. doi: 10.1186/s13044-024-00202-x (PMC11292864; doi:10.1186/s13044-024-00202-x)
Supplement: Supplementary file 1 — Supplementary Material 1 [file 13044_2024_202_MOESM1_ESM.docx]

**Additional file 1**

**Table S1.** Transfection efficiency (%) in 6-well plate containing 8×10⁵ cells.

| **Plasmid DNA (µg)** | **transfection efficiency** (%) | **Time**  **(hour)** |
| --- | --- | --- |
| 2 | 41.3 | 48 |
| 4 | 54.2 | 48 |
| 6 | 69.7 | 48 |
| 7 | 71.9 | 48 |
| 2 | 49.5 | 72 |
| 4 | 67.2 | 72 |
| 6 | 86.6 | 72 |
| 7 | 87.7 | 72 |
